# Genome-Wide DNA Methylation Analysis Identifies Novel Hypomethylated Non-Pericentromeric Genes with Potential Clinical Implications in ICF Syndrome

**DOI:** 10.1371/journal.pone.0132517

**Published:** 2015-07-10

**Authors:** L. Simo-Riudalbas, A. Diaz-Lagares, S. Gatto, M. Gagliardi, A. B. Crujeiras, M. R. Matarazzo, M. Esteller, J. Sandoval

**Affiliations:** 1 Cancer Epigenetics and Biology Program (PEBC), Bellvitge Biomedical Research Institute (IDIBELL), Barcelona, Catalonia, Spain; 2 Institute of Genetics and Biophysics ABT, CNR, Naples, Italy; 3 Department of Physiological Sciences II, School of Medicine, University of Barcelona, Barcelona, Catalonia, Spain; 4 Institucio Catalana de Recerca i Estudis Avançats (ICREA), Barcelona, Catalonia, Spain; Institut National de la Santé et de la Recherche Médicale, FRANCE

## Abstract

**Introduction and Results:**

Immunodeficiency, centromeric instability and facial anomalies syndrome (ICF) is a rare autosomal recessive disease, characterized by severe hypomethylation in pericentromeric regions of chromosomes (1, 16 and 9), marked immunodeficiency and facial anomalies. The majority of ICF patients present mutations in the DNMT3B gene, affecting the DNA methyltransferase activity of the protein. In the present study, we have used the Infinium 450K DNA methylation array to evaluate the methylation level of 450,000 CpGs in lymphoblastoid cell lines and untrasformed fibroblasts derived from ICF patients and healthy donors. Our results demonstrate that ICF-specific DNMT3B variants A603T/STP807ins and V699G/R54X cause global DNA hypomethylation compared to wild-type protein. We identified 181 novel differentially methylated positions (DMPs) including subtelomeric and intrachromosomic regions, outside the classical ICF-related pericentromeric hypomethylated positions. Interestingly, these sites were mainly located in intergenic regions and inside the CpG islands. Among the identified hypomethylated CpG-island associated genes, we confirmed the overexpression of three selected genes, BOLL, SYCP2 and NCRNA00221, in ICF compared to healthy controls, which are supposed to be expressed in germ line and silenced in somatic tissues.

**Conclusions:**

In conclusion, this study contributes in clarifying the direct relationship between DNA methylation defect and gene expression impairment in ICF syndrome, identifying novel direct target genes of DNMT3B. A high percentage of the DMPs are located in the subtelomeric regions, indicating a specific role of DNMT3B in methylating these chromosomal sites. Therefore, we provide further evidence that hypomethylation in specific non-pericentromeric regions of chromosomes might be involved in the molecular pathogenesis of ICF syndrome. The detection of DNA hypomethylation at BOLL, SYCP2 and NCRNA00221 may pave the way for the development of specific clinical biomarkers with the aim to facilitate the identification of ICF patients.

## Introduction

The immunodeficiency, centromeric instability and facial anomalies syndrome (ICF) is a rare recessive disorder, with less than 60 cases reported worldwide. ICF syndrome is characterized by two peculiar signs: a variable immunodeficiency and a recurrent instability of pericentromeric heterochromatin, which usually leads to chromosome breakage in mitogen-stimulated lymphocytes. The chromosomal abnormalities are found exclusively in hypomethylated pericentromeric regions of chromosome 1, 16 and less frequently in 9. Other ICF symptoms count in facial anomalies, psychomotor and mental retardation and developmental delay [1].

The importance of ICF pathology, at the molecular level, relies on the fact that it is the only human disease showing mendelian inheritance of aberrant DNA methylation, caused by mutations in one of the three main DNA-methyltransferase genes, DNMT3B. Approximately 50% of the ICF cases, defined as ICF type1, present biallelic DNMT3B mutations located mainly in the catalytic domain of the protein, often leading to the impairment of its methyltransferase activity [2]. Among the rest of patients some carry nonsense mutations in zinc-finger and BTB domain-containing 24 gene (ZBTB24), designated as ICF2 patients, while a small group of them has still unknown etiology, and are designated as ICFX [2].

The biochemical defects in DNMT3B-mediated de novo DNA methylation have been recently assessed by in vitro studies of the ICF-associated DNMT3B variants [3]. These results reveal that catalysis by DNMT3B is much more complex than expected. In that context, ICF mutations cause a broad spectrum of biochemical defects in DNMT3B function, including defects in homo-oligomerization, SAM binding, SAM utilization and DNA binding [3].

Although it is well established in literature that all ICF1 patient derived cells exhibit targeted DNA hypomethylation of the pericentromeric heterochromatin at chromosomes 1, 16 and sometimes 9, the molecular defects in global DNA methylation caused by ICF-specific DNMT3B mutants remain relatively uncharacterized. Subtle differences in local DNA methylation patterns have been undetected by less sensitive assays previously employed, such as two-dimensional gel electrophoresis of methylation-sensitive digested genomic DNA and COBRA analysis [4],[5]. Recently, several studies suggested that DNA hypomethylation might be more extended than previously thought [6][7]. In this sense, previous genome-wide work from our laboratory, detected DNA hypomethylation in the inactive heterochromatic regions, satellite repeats and transposons associated to two heterozygous DNMT3B mutations in one ICF1 patient [8].

In this study, a state-of-the-art DNA-methylation high-resolution microarray (*HumanMethylation450k BeadChip)* from Illumina (San Diego, CA) that interrogates 450,000 CpGs sites in the human genome was used to find out the impact of four different ICF-specific DNMT3B mutant alleles (A603T/STP807ins and V699G/R54X) on DNA methylation at a global level. This will help us elucidate and understand the potential pivotal role of this heterogeneous epigenetic mechanism in ICF syndrome pathogenesis. The identification of potential biomarkers for ICF patients after validation in peripheral blood samples of ICF patients might allow us to design more effective strategies to address the diagnosis or treatment of this disease.

## Materials and Methods

### Sample preparation

The current analysis was performed by evaluating one lymphoblastoid (LCL) cell line and untrasformed fibroblasts derived from two different ICF patients with compound and different heterozygous mutations in DNMT3B gene (PT5 and GM08747) compared to LCLs (XX and MS) and fibroblasts (3674) derived from 3 unrelated controls. PT5 sample comes from an ICF male with heterozygous DNMT3B mutations (V699G/R54X). GM08747 comes from a female diagnosed for ICF syndrome with heterozygous DNMT3B mutations (A603T/STP807ins). The healthy donors samples belonged to two females (XX, GM03674) and one male (MS). All of these cell lines were obtained from Dr. RS Hansen laboratory (University of Washington, USA) and the Coriell Cell Repository and have been used in a previous published article [9]. Additionally, DNA from mononuclear cells isolated from four cord blood donors (three males and one female) was kindly provided by Dr. D. Monk from PEBC (IDIBELL, Spain). For validation steps, we extended the cohort with three lymphoblastoid cell lines: GM08714 from the ICF female patient with mutations A603T/STP807ins (see above) and its related healthy donors (GM08728 and GM08729), identified as the GM08714’s mother (GM08728) and father (GM08729) and another primary fibroblast PT3 from the ICF male with heterozygous DNMT3B mutations (V699G/R54X). Another unrelated LCL control was used (LDA). All these cell lines were also obtained and purchased from the Coriell Cell Repository. The project has been approved by the local Ethical Committee of the IDIBELL Institution.

### Genome-wide DNA methylation analysis

Genome-wide DNA methylation analysis was performed using the Infinium HumanMethylation450 BeadChip from Illumina. The 450K DNA methylation array by Illumina is an established, highly reproducible method for DNA methylation detection and has been validated in two independent laboratories [10],[11].

DNA from ICF patients and healthy donors were isolated using Phenol:Chloroform:Isoamylalcohol (Sigma) and quantified by Quant-iT PicoGreen dsDNA Reagent (Invitrogen). The integrity was analyzed in a 1.3% agarose gel. Bisulfite conversion of 600 ng of each DNA sample was performed according to the manufacturer's recommendation for Illumina Infinium Assay. Effective bisulfite conversion was checked for three controls that were converted simultaneously with the samples. Four μl of bisulfite converted DNA were used to hybridize on Infinium Human Methylation 450 BeadChip, following Illumina Infinium HD Methylation protocol. Chip analysis was performed using Illumina HiScan SQ fluorescent scanner. The intensities of the images were extracted using GenomeStudio (2010.3) Methylation module (1.8.5) software (San Diego, California). Methylation score of each CpG is represented as beta (β) value. The 450K DNA Methylation array includes 485,764 cytosine positions of the human genome that were filtered by sex chromosomes CpGs (avoiding sex link alterations) and non-valid CpGs (p-value<0.001). The intensities of the images were extracted and normalized using GenomeStudio (2011.1) Methylation module (1.9.0) software. Unsupervised (using the 5000 random CpGs) and supervised heatmaps were obtained using hierarchical clustering analysis with Manhattan metrics.

For determining differentially methylated CpGs a parametric analysis using an absolute difference in beta values of 0.65 and standard deviation <0.15 in ICF1 patients compared to controls were used for selecting the most relevant positions.

### Bisulfite genomic DNA sequencing

The Methyl Primer Express v1.0 software (Applied Biosystems, Life technologies, Grand Island, New York) was used to identify the CpG islands in gene promoter regions and to design specific primers for the methylation analysis (S1 Table). DNA methylation status was established by bisulfite genomic sequencing. DNA was extracted from samples using the DNeasy tissue kit (Qiagen, Milan, Italy) and 1 μg of DNA was modified with sodium bisulfite using the EZ DNA methylation-gold kit (Zymo Research, CA USA) according to manufacturer´s instructions. Multiple clones were analyzed for each sample and the methylation frequency was calculated in each case.

### Gene expression analysis

Total RNA from lymphoblastoid cell lines and untrasformed fibroblasts was extracted using TRIzol reagent (Life Technologies. Grand Island, New York) and was reverse-transcribed using iScript cDNA Synthesis kit (Bio-rad). Quantitative real-time PCR (qRT-PCR) was performed using iQ Supermix SYBR Green 2X (Bio-rad. San Diego, California) on the Bio-Rad iCycler according to the manufacturer’s protocols. The ΔΔCt method [12] was used to determine relative quantitative levels. GAPDH gene was used to normalize the data. Primer sequences for gene expression analysis are shown in (S2 Table). Statistical analysis was performed using Student t test.

## Results and Discussion

### ICF-specific DNMT3B variants cause a global decrease in DNA methylation profile

Seminal studies regarding DNA methylation in ICF1 patients have reported significant hypomethylation at pericentromeric satellite DNA sequences in ICF cells of chromosomes 1, 9, 16, Alu sequences, D4Z4 and NBL2 repeats. Heterogeneous hypomethylation has been described at few single copy loci, X-linked and imprinted genes, while the genomic hypomethylation in ICF1 patients has been thought to be involved only in a rather small percentage of the 5- methylcytosine residues [4],[13–17]. Recently, a genome-wide DNA methylation analysis has been performed in our laboratory with the limitation of using a unique patient sample [8].

**Fig 1:**
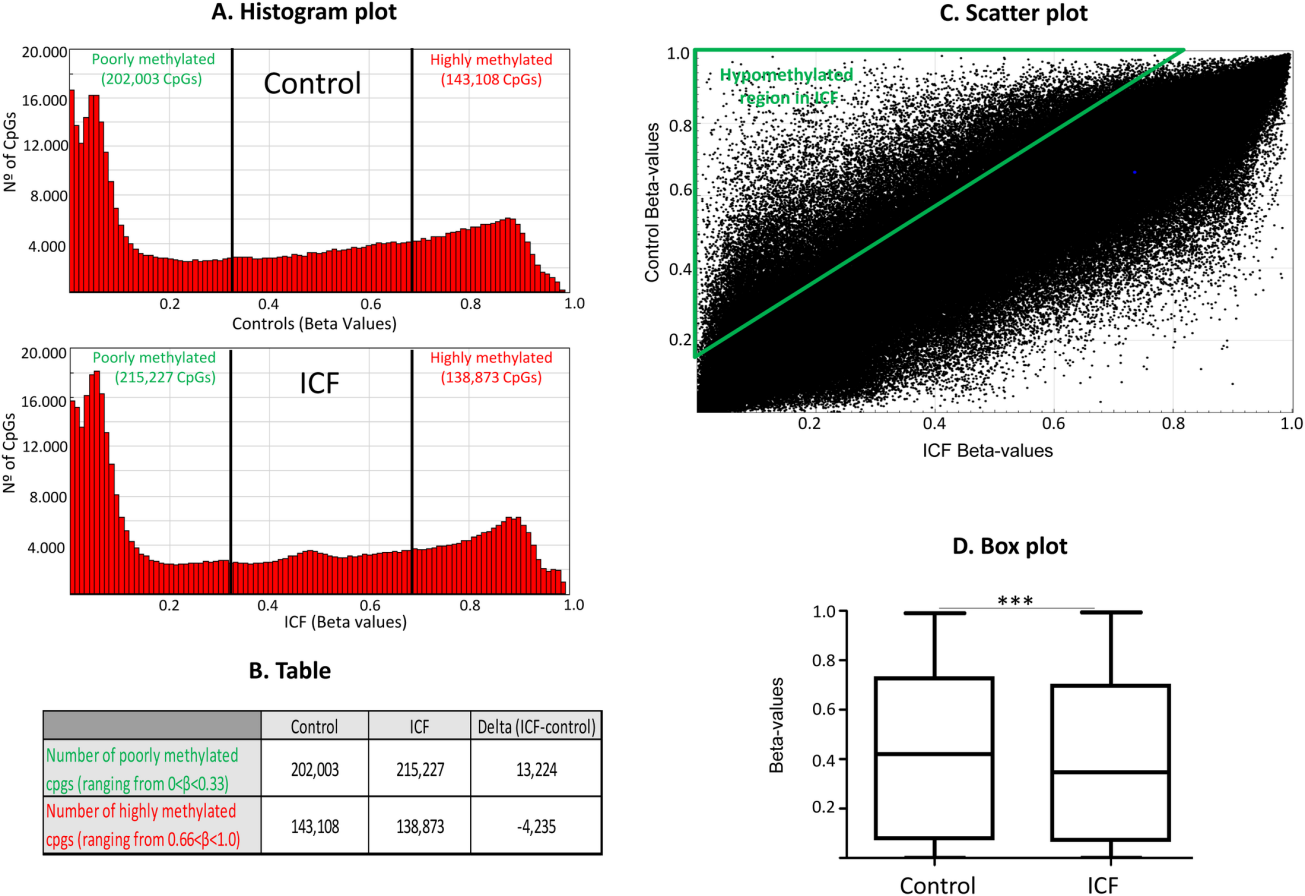
Genome-wide DNA methylation profiles in ICF patients and control samples. (A) Histograms shows bimodal distribution pattern of DNA methylation profiles in ICF patients and normal donors. The frequency of CpGs according to DNA methylation levels are depicted in the graph. (B) Table showing number of average poorly methylated (methylation levels beta<0.33) and average highly methylated (methylation levels Beta>0.66). (C) Scatter plot represents comparison of DNA methylation levels of total CpG sites using the Infinium 450K DNA methylation assay. Green triangle selects hypomethylated area for ICF patients compared to controls. (D) Box plot displaying the distribution of Beta-values of total CpG sites of ICF *versus* healthy control donors. Normality was tested using the Shapiro-Wilk test and significance was evaluated with the Mann-Whitney U test and is indicated by three asterisks *** (p<0.001).

Since the majority of ICF patients deal with mutations within the DNMT3B catalytic domain expected to variably interfere with the methyltransferase activity of the protein, the primary goal of this study was to describe the global DNA methylation profile affected by ICF specific DNMT3B mutant alleles. Here, we characterized the methylome of one lymphoblastoid cell line and of untrasformed fibroblasts derived from two different compound heterozygous ICF patients with the DNMT3B mutations V699G/R54X and A603T/STP807ins (PT5 and GM08747, respectively) compared to three control LCLs (XX, MS) and fibroblasts 3674 derived from healthy donors. Using this strategy that includes ICF patients derived from different tissue types, the variability and interference due to tissue-specific genes, will be reduced. The analysis, by calculating first the averaged Beta values for each CpG from the three controls and average Beta values from the two ICF patients and later the delta values (average ICF-average controls), shows that ICFs globally contain more poorly methylated (βvalue<0.33) and less highly methylated CpGs (βvalue>0.66) compared to controls (Fig 1A). In this sense, the accumulated number of poorly methylated CpGs ranging Beta values from 0 to 0.33 of ICF patients is 215,227; while for controls decrease to 202,003 (Delta ICF-Control = +13,224). However, an opposite pattern is obtained for highly methylated CpGs ranging from Beta values 0.66 to 1. In this case, control donor showed 143,108 highly methylated CpGs compared to lower number for ICF patients 138,873 CpGs (Delta ICF-Control = -4,235) (Fig 1B). A more comprehensive representation is the scatter plot of the DNA methylation levels (βvalue) of ICF patients compared to controls showing a higher accumulation of hypomethylated CpGs in ICFs than in controls, see triangle area in Fig 1C. Confirming these results we observed, using a non-parametic Mann-withney U test after testing normality with the Shapiro-Wilk test, a significant decrease in methylation level in ICF samples compared to controls (Fig 1D). We provide individual histograms, scatter and box plots for all the hybridized samples. Individual methylation levels were consistent, although control 2 (XX) presented lower global levels than the other two controls (MS and GM03674) (S1 Fig). Therefore, our results are in agreement with previous studies reporting that ICF syndrome is a disease characterized by DNA hypomethylation and we further demonstrate that the combination of the specific DNMT3B variants A603T/STP807ins and V699G/R54X derives in a global loss of DNA methylation levels.

### Identification of differentially DNA methylated genes in ICF

Previous studies, using candidate-gene approaches, have been searching for differentially methylated regions in ICF patients that would account for the severe clinical features that characterize the ICF patients. In this sense, Jin *et al* reported subtle but significant changes in DNA methylation levels associated with transcript level variations in a few genes involved in development, neurogenesis and immunological function of ICF patients using expression microarrays [6]. Moreover, high degree correlation between DNA methylation changes and gene expression patterns for a number of heterochromatic genes located at the pericentromeric region of chromosome 21 in ICF patients has been also reported [7]. Therefore, a genome-scale approach would contribute to identify new differentially methylated genes and increase the knowledge in the etiology and development of the disease.

**Fig 2:**
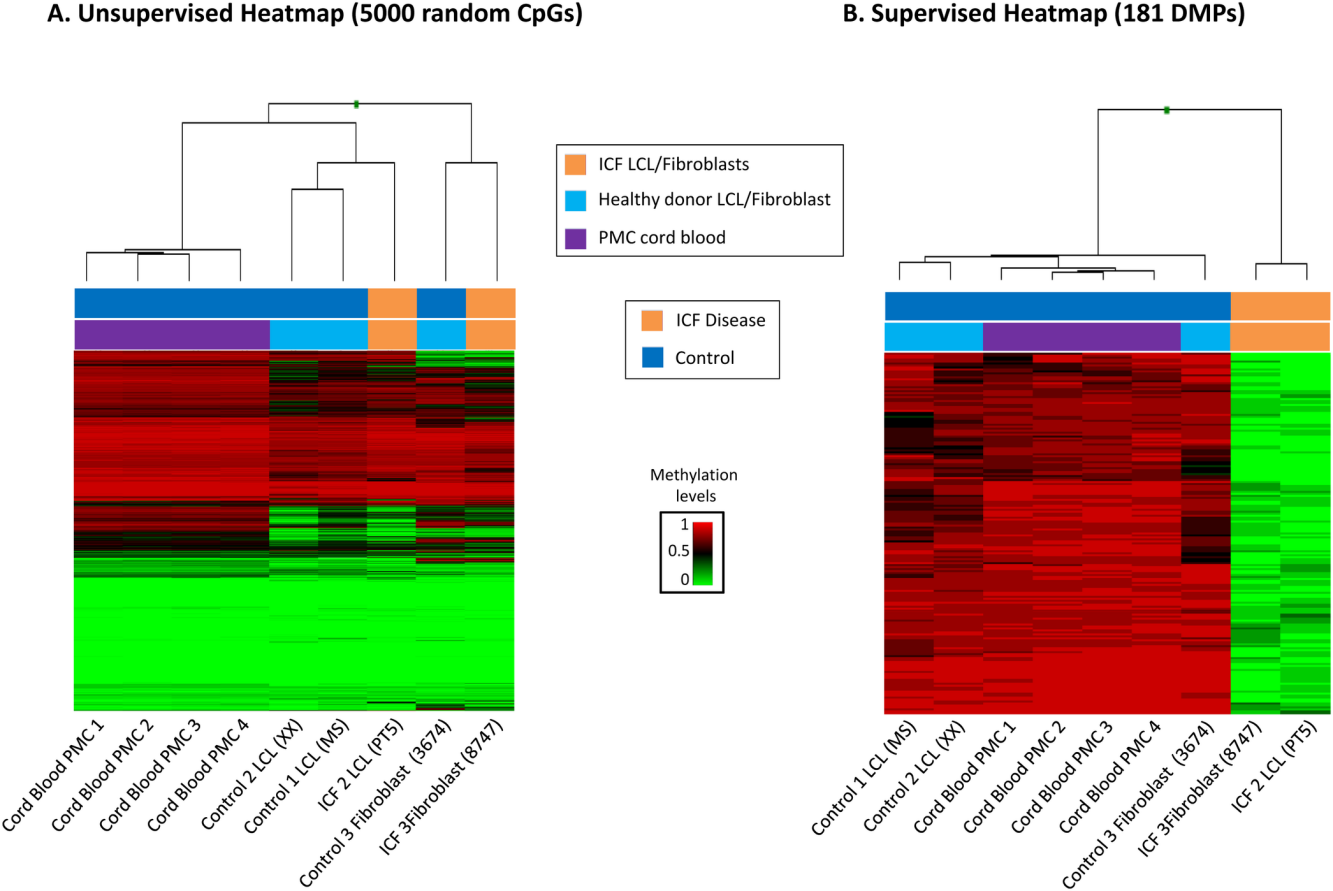
Identification of Differentially methylated CpGs. (A) Unsupervised hierarchical clustering and heatmap of four cord blood donors (purple), three unrelated healthy donors (blue) and two ICF patients (orange) using 5000 random selected CpGs. DNA Methylation levels scale is shown. Each column represents patients and each row represents the different CpGs. (B) Supervised cluster and heatmap representing the distinctive 181 CpGs corresponding to the comparison between ICF patients (orange) and all control samples (dark blue).

Since we observed that ICF patients with DNMT3B mutations show a global reduction of DNA methylation, we focused on studying the CpG positions with loss of methylation. To gain robustness and reliability, we added to the previous set of ICF1 samples four new controls of peripheral mononuclear cells obtained from cord blood samples (CB10, CB13, CB20 and CB76), being aware of the limitation of the cell type heterogeneity in these samples. The unsupervised hierarchical clustering, using 5000 random CpGs mimicking the global methylome, shows that samples are grouped based on their tissue type. The methylome of the four peripheral mononuclear derived cells samples is homogeneous and clustered together. The immortalized cell lines derived from controls and ICF patients clusterized in the same group, but in a separate subgroup. Finally, fibroblast cells clustered in an independent group (Fig 2A). These results indicate that the distance between samples is mainly due to tissue type.

We focused on ICF-associated regions which had loss of methylation, in order to obtain reliable and ICF-specific differentially methylated candidates. To achieve this, we used a restrictive threshold to overcome the limited number of samples and the heterogeneous effect of the mutations. Then, we performed a parametric analysis comparing average Beta values from ICF1 patients versus controls selecting those with differences in methylation levels higher than 65% (delta<-0.65) and a standard deviation value lower than 15% (Desvest<0.15). Using this strategy, with restrictive cut-offs, we identified 181 hypomethylated CpGs in ICF1 patients compared to control group. A table with the 181 differentially methylated positions (DMPs) with their gene characteristics including target ID, name and Beta values for ICF1 and controls groups, chromosome location, genomic distribution and CpG context is provided (Table 1). The hierarchical clustering heatmap for all these selected 181 CpG sites clearly segregates both groups (Fig 2B). It is worth emphasizing that both ICF1 patients present different tissue types and DNMT3B mutations, thereby producing subtle distinct levels of DNA methylation defects. However, the selected approach allowed us to identify the epigenetic differences that are common to both ICF1 patients leading to the establishment of potential markers for the ICF1 disease. Furthermore, we aimed to compare our selected 181 DMPs with the previous published methylome in our laboratory by Heyn *et al*. of the LCL GM08714 from the same ICF patient from which the GM08747 fibroblasts derive [8]. Using whole genome bisulfite sequencing (WGBS) we previously obtained 296,964 differentially methylated regions (DMRs). Consistently, we observed that a high number of 138 out of 181 (76.2%) of DMPs were inside a previously defined DMRs and therefore are common to both studies (S2A Fig). It is important to emphasize that although we are using different ICF1 samples from the previously published work, there is a high percentage of concordance that favors the reliability of our results.

**Table 1:** List of Differentially Methylated CpGs comparing ICF patients and healthy controls.

TargetID	average(controls)	average(ICF)	Delta(ICF- controls)	CHR	MAPINFO	Name	Genomicdistribution	CpGisland
cg03986562	0.84	0.03	-0.82	16	7703893	A2BP1	Body	Island
cg12799314	0.76	0.07	-0.69	10	1405193	ADARB2	Body	Island
cg01962727	0.86	0.14	-0.73	16	46603059	ANKRD26P1	TSS200	
cg07121021	0.85	0.18	-0.67	10	37414597	ANKRD30A	TSS200	Island
cg00381789	0.88	0.17	-0.71	11	424667	ANO9	Body	Shelf
cg01669186	0.95	0.20	-0.75	11	430919	ANO9	Body	Island
cg26217813	0.85	0.13	-0.72	17	3378996	ASPA	5'UTR	Shelf
cg10204707	0.95	0.10	-0.85	19	53794711	BIRC8	5'UTR	Island
cg00361562	0.92	0.08	-0.84	2	198649771	BOLL	5'UTR	Island
cg10547527	0.94	0.08	-0.86	2	198650123	BOLL	TSS200	Island
cg13379983	0.94	0.12	-0.82	2	198649727	BOLL	5'UTR	Shore
cg21300403	0.93	0.09	-0.84	2	198650112	BOLL	TSS200	Island
cg00982548	0.92	0.08	-0.84	2	198649783	BOLL	5'UTR	Island
cg11031976	0.85	0.18	-0.67	2	198649780	BOLL	5'UTR	Island
cg04006718	0.88	0.19	-0.69	10	1068667	C10orf110	Body	
cg05820861	0.77	0.04	-0.73	13	24882296	C1QTNF9	TSS1500	Island
cg18167759	0.92	0.27	-0.65	12	2017746	CACNA2D4	Body	Island
cg06839970	0.89	0.10	-0.79	11	15093769	CALCB	TSS1500	Shore
cg03749198	0.84	0.12	-0.72	19	51979766	CEACAM18	TSS200	
cg07369559	0.78	0.10	-0.69	16	71559445	CHST4	TSS1500	
cg17754473	0.87	0.12	-0.75	4	798611	CPLX1	Body	Island
cg12517410	0.75	0.09	-0.66	2	115612096	DPP10	Body	
cg03272326	0.74	0.08	-0.65	1	166136939	FAM78B	TSS1500	Shore
cg01476885	0.77	0.12	-0.65	17	21908498	FLJ36000	Body	
cg02432860	0.88	0.21	-0.67	22	17489187	GAB4	TSS200	Island
cg00028598	0.91	0.12	-0.79	15	27128633	GABRA5	Body	Island
cg17403731	0.76	0.06	-0.70	19	613505	HCN2	Body	Island
cg26645432	0.83	0.13	-0.70	6	33048502	HLA-DPB1	Body	Island
cg21332305	0.84	0.11	-0.73	6	32729181	HLA-DQB2	Body	Shore
cg19831369	0.89	0.10	-0.79	22	26881026	HPS4	TSS1500	Shore
cg18515063	0.80	0.14	-0.66	17	61616209	KCNH6	Body	Shore
cg11195059	0.80	0.09	-0.71	7	107741042	LAMB4	Body	
cg11850943	0.79	0.10	-0.70	7	55432402	LANCL2	TSS1500	Shore
cg15463284	0.76	0.07	-0.68	11	18477534	LDHAL6A	5'UTR	Island
cg03003467	0.88	0.05	-0.83	14	106938289	LOC100133469	TSS200	Island
cg14404807	0.92	0.07	-0.86	14	106938294	LOC100133469	TSS200	Island
cg16023283	0.82	0.04	-0.78	14	106938234	LOC100133469	TSS1500	Island
cg26117778	0.93	0.09	-0.85	14	106938335	LOC100133469	TSS200	Island
cg17347810	0.84	0.16	-0.68	19	42349010	LYPD4	TSS1500	Shore
cg08247921	0.90	0.18	-0.72	4	103683115	MANBA	TSS1500	Shore
cg01320920	0.90	0.23	-0.67	3	12597236	MKRN2	TSS1500	Shore
cg05013438	0.86	0.18	-0.68	8	125631251	MTSS1	Body	
cg26954695	0.89	0.10	-0.80	19	59085561	MZF1	TSS1500	Shore
cg26747317	0.74	0.05	-0.70	6	31829793	NEU1	Body	Shore
cg18438823	0.75	0.08	-0.67	11	4676365	OR51E1	3'UTR	
cg16599007	0.89	0.19	-0.70	12	121647205	P2RX4	TSS1500	Shore
cg14995055	0.90	0.09	-0.82	5	140240593	PCDHs	Body	Shore
cg00635356	0.71	0.05	-0.66	5	140603942	PCDHB14	1stExon	Shore
cg24403041	0.80	0.15	-0.66	21	14982363	POTED	TSS200	
cg08691323	0.84	0.04	-0.80	5	54826683	PPAP2A	Body	Island
cg16411279	0.94	0.02	-0.92	5	54826768	PPAP2A	Body	Island
cg03473125	0.96	0.25	-0.72	7	157550806	PTPRN2	Body	Island
cg22386389	0.98	0.13	-0.85	2	238707268	RBM44	TSS200	Island
cg00399591	0.86	0.21	-0.65	16	18802159	RPS15A	TSS1500	Shore
cg27258551	0.84	0.07	-0.77	16	23197740	SCNN1G	Body	Island
cg13924635	0.95	0.08	-0.87	1	1168432	SDF4	TSS1500	Island
cg21396646	0.76	0.10	-0.66	1	234039761	SLC35F3	TSS1500	Shore
cg04963199	0.81	0.11	-0.69	16	87868947	SLC7A5	Body	
cg10700435	0.85	0.15	-0.71	1	40838541	SMAP2	TSS1500	Shore
cg14608581	0.75	0.02	-0.73	2	70122240	SNRNP27	Body	
cg10460168	0.82	0.10	-0.72	5	112198022	SRP19	Body	Shore
cg11258452	0.83	0.04	-0.79	12	119594454	SRRM4	Body	Shelf
cg16266492	0.77	0.09	-0.68	10	135379461	SYCE1	5'UTR	Island
cg16654419	0.90	0.09	-0.81	10	135379580	SYCE1	5'UTR	Island
cg24678803	0.81	0.13	-0.68	10	135378570	SYCE1	5'UTR	Shore
cg21950459	0.97	0.06	-0.91	20	58508076	SYCP2	TSS1500	Island
cg02820072	0.92	0.12	-0.79	19	33210552	TDRD12	TSS200	Island
cg18155407	0.92	0.11	-0.81	19	33210662	TDRD12	TSS200	Island
cg19712189	0.79	0.11	-0.68	19	33210851	TDRD12	5'UTR	Island
cg12580503	0.89	0.18	-0.72	12	130340287	TMEM132D	Body	Island
cg03862437	0.87	0.11	-0.76	3	194353432	TMEM44	Body	Shore
cg02203665	0.75	0.06	-0.68	9	100262506	TMOD1	TSS1500	Shore
cg25691239	0.81	0.14	-0.68	21	10991412	TPTE	TSS1500	Island
cg09612946	0.84	0.16	-0.68	2	220121680	TUBA4B	Body	Shelf
cg02359511	0.81	0.11	-0.70	4	186346156	UFSP2	Body	Shore
cg13913666	0.79	0.13	-0.66	7	1198122	ZFAND2A	5'UTR	Shore
cg00469395	0.90	0.17	-0.73	16	89315104			Island
cg00654159	0.80	0.10	-0.70	16	32858241			Island
cg00813343	0.83	0.14	-0.68	7	109485219			
cg00964333	0.89	0.05	-0.83	10	15038045			
cg01086526	0.88	0.16	-0.72	5	3188191			Island
cg01215328	0.92	0.10	-0.82	16	34208792			Island
cg01390039	0.93	0.22	-0.71	16	89315293			Island
cg01403596	0.89	0.10	-0.79	2	92045973			
cg01873249	0.88	0.23	-0.65	16	34442602			Shore
cg01893629	0.86	0.09	-0.77	12	34494825			Island
cg02052758	0.90	0.18	-0.72	7	55430948			Shelf
cg02491199	0.96	0.07	-0.90	17	21901968			
cg02506248	0.83	0.10	-0.73	12	130502267			
cg02587606	0.77	0.10	-0.67	10	77352231			
cg02797144	0.81	0.07	-0.74	16	3245099			Shore
cg02801570	0.85	0.13	-0.72	10	15026284			
cg02811702	0.87	0.08	-0.78	13	24901961			Shore
cg02985366	0.81	0.14	-0.67	16	32360428			Island
cg03563298	0.86	0.07	-0.79	2	242990263			Shore
cg03961510	0.87	0.07	-0.80	10	2978438			
cg04523661	0.94	0.22	-0.72	19	1302808			Island
cg04696808	0.90	0.13	-0.77	16	34257170			Island
cg04815577	0.72	0.03	-0.69	5	51898			Island
cg05136737	0.81	0.12	-0.69	1	244924918			
cg05507257	0.86	0.17	-0.69	10	135524081			
cg05585551	0.87	0.09	-0.78	6	167314745			Island
cg06064212	0.80	0.13	-0.67	6	28455048			Shelf
cg06217323	0.72	0.04	-0.67	3	75445502			Island
cg06446408	0.83	0.12	-0.71	16	73266679			
cg06766960	0.79	0.08	-0.71	11	133703094			Island
cg06953577	0.84	0.13	-0.70	11	113659952			Shore
cg07088555	0.93	0.07	-0.86	16	89315186			Island
cg08198040	0.78	0.09	-0.69	16	34429930			Shore
cg08363684	0.89	0.12	-0.77	6	761547			
cg08377526	0.82	0.13	-0.70	2	92261659			Island
cg08617346	0.82	0.13	-0.69	20	1716331			Island
cg08761055	0.93	0.08	-0.85	2	5846917			Island
cg08767686	0.77	0.01	-0.76	5	51426			Island
cg08860346	0.82	0.09	-0.73	2	75504008			
cg09415274	0.86	0.17	-0.69	15	40422219			
cg09605095	0.79	0.04	-0.76	2	5847414			Island
cg09785512	0.78	0.09	-0.69	17	21934840			Island
cg09817162	0.75	0.03	-0.72	6	27185676			
cg09871679	0.87	0.08	-0.79	7	61821180			Island
cg10399005	0.78	0.07	-0.71	14	70316898			Island
cg10606029	0.86	0.20	-0.65	5	37952598			
cg10854819	0.75	0.02	-0.72	15	31515852			
cg11115267	0.84	0.15	-0.68	10	38908948			
cg11350504	0.77	0.09	-0.68	13	113121280			Shore
cg11476737	0.84	0.07	-0.76	10	2815189			Island
cg11837817	0.90	0.09	-0.81	6	167314233			Island
cg11860886	0.93	0.07	-0.85	14	106857312			Island
cg12109260	0.83	0.06	-0.77	8	144850435			Shelf
cg12161959	0.84	0.19	-0.65	2	240868337			Island
cg12492942	0.89	0.18	-0.71	6	27510966			
cg12647837	0.91	0.16	-0.75	7	61820842			Island
cg13396152	0.87	0.19	-0.67	1	62005072			
cg14026325	0.93	0.02	-0.90	8	144787867			Island
cg14218861	0.77	0.08	-0.70	10	119443973			
cg14303972	0.84	0.05	-0.79	12	34752640			
cg14341968	0.85	0.19	-0.66	9	136075868			Shore
cg14493094	0.79	0.10	-0.68	4	132896579			Island
cg15335669	0.77	0.05	-0.72	15	22546908			Island
cg15646803	0.77	0.12	-0.65	2	92261070			Island
cg16530595	0.81	0.07	-0.74	8	144788492			Island
cg17024593	0.75	0.06	-0.68	12	34490115			Island
cg18304339	0.83	0.16	-0.67	14	52213734			
cg18315943	0.79	0.12	-0.67	5	3188374			Island
cg18480548	0.87	0.09	-0.78	19	523642			Island
cg18686665	0.87	0.06	-0.81	2	629121			Island
cg18709881	0.75	0.07	-0.69	18	72837627			Island
cg18710908	0.83	0.18	-0.65	8	144787999			Island
cg18898632	0.80	0.14	-0.66	2	242989856			Island
cg18963811	0.81	0.14	-0.66	14	52210597			
cg18969117	0.95	0.13	-0.81	1	226269917			Shore
cg19035254	0.87	0.17	-0.70	8	43131260			Island
cg19443750	0.73	0.08	-0.65	13	112996588			Shore
cg19766591	0.81	0.12	-0.69	10	2978190			
cg20301678	0.90	0.14	-0.76	6	167313267			Island
cg20447730	0.84	0.06	-0.79	10	45719880			Island
cg21053539	0.89	0.20	-0.69	6	28829503			Shelf
cg21451653	0.76	0.07	-0.68	12	34551994			
cg21561672	0.89	0.19	-0.70	14	102101551			Island
cg21918786	0.90	0.10	-0.80	6	109611834			Island
cg21941223	0.95	0.14	-0.81	19	2926894			Island
cg21973687	0.82	0.12	-0.70	6	148142629			
cg22243348	0.84	0.13	-0.71	19	11874227			Shelf
cg22778503	0.90	0.09	-0.82	14	106857188			Island
cg22834924	0.83	0.09	-0.74	6	140392531			
cg22973226	0.90	0.21	-0.69	1	46705909			Island
cg23193831	0.83	0.11	-0.72	11	43177729			
cg23207305	0.83	0.12	-0.71	11	15012901			
cg23771956	0.73	0.03	-0.70	10	45720040			Island
cg23972271	0.87	0.21	-0.66	5	98541824			
cg23997132	0.86	0.02	-0.84	10	15038349			
cg24854181	0.80	0.02	-0.78	5	6823241			Island
cg25606780	0.87	0.20	-0.68	3	47397852			Island
cg25854527	0.77	0.08	-0.69	3	102333494			
cg26427818	0.96	0.12	-0.84	11	15093613			Shore
cg26931296	0.86	0.17	-0.69	7	150870852			Island
cg27399125	0.78	0.05	-0.72	6	150378738			Shore
cg27427743	0.78	0.13	-0.65	13	23270850			Island
cg27438152	0.80	0.08	-0.72	15	31515761			
cg27629454	0.80	0.14	-0.66	1	228742687			Shore
cg27660756	0.82	0.08	-0.74	19	57103802			Shelf

### Characterization of the genomic localization and gene features of obtained DMPs

**Fig 3:**
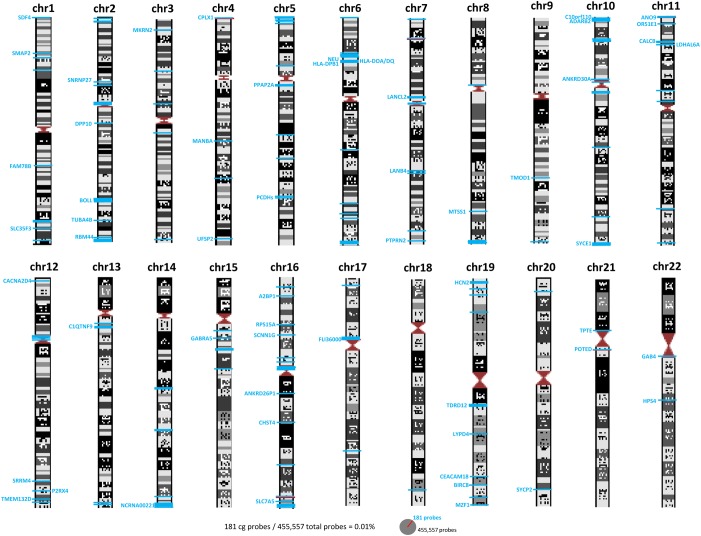
Schematic representation of chromosomes and CpG localization. Gene names and intergenic CpGs are represented and localized by blue lines.

**Fig 4:**
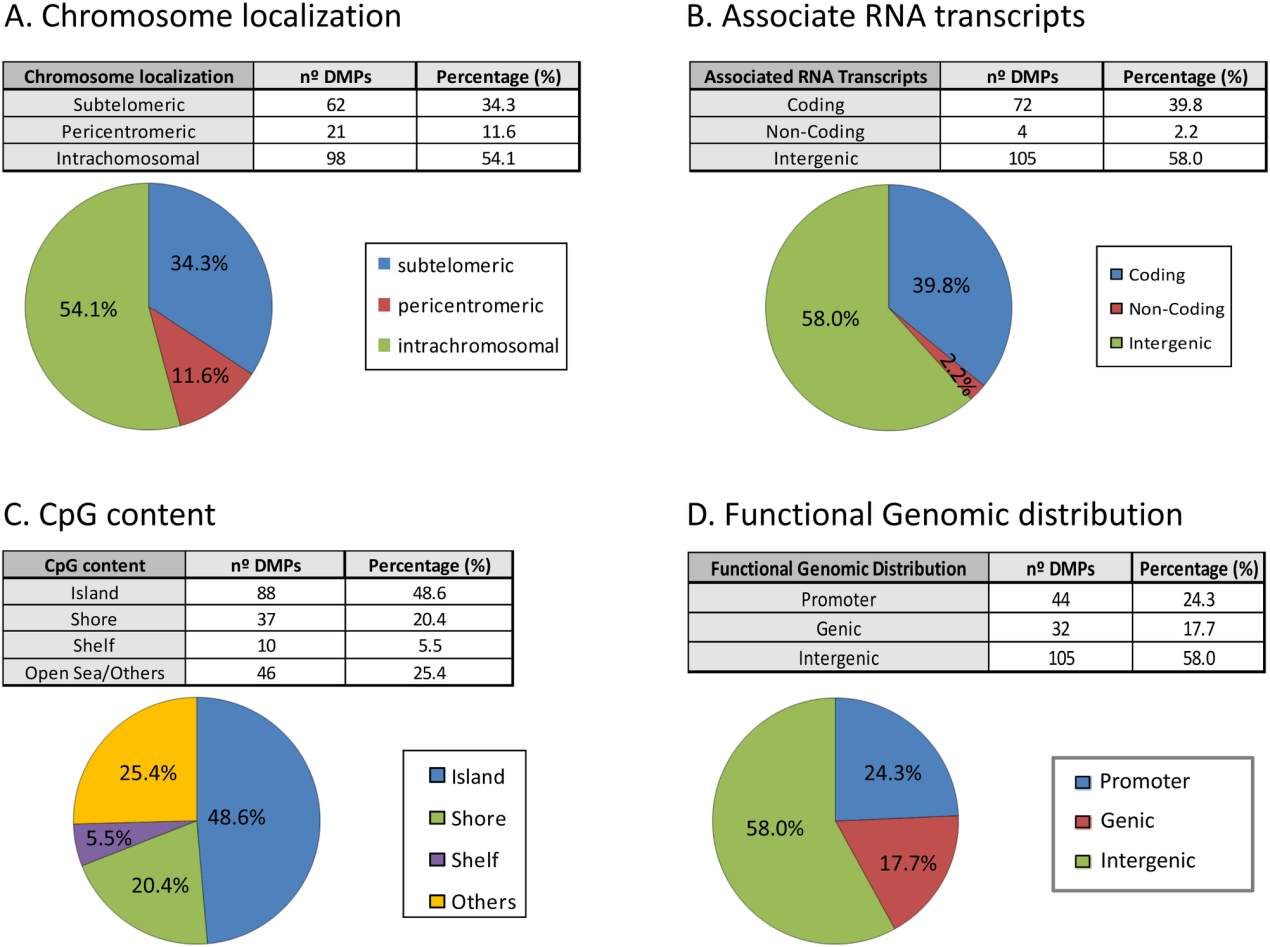
Genomic distribution and gene features of the 53 differentially methylated CpGs. (A) Chromosomal sub-localization classified in different groups: subtelomeric, pericentromeric and intrachromosomal. (B) Associated RNA transcription classified in: coding, non-coding and intergenic. (C) CpG context and neighborhood classified in: island, shore, shelf and open sea/others. (D) Functional genomic distribution classified in: promoter (TSS1500, TSS200, 5´UTR), genic (1^st^exon, body and 3´UTR) and intergenic.

ICF-specific DNA methylation changes have been previously reported mainly in pericentromeric regions of chromosomes 1, 9 and 16 [18]. However, when we analyzed the subchromosomal localization of our DMPs we observed that the majority of the DMPs are located in the intra and subtelomeric regions of the chromosomes (Figs 3 and 4A). These results support previous studies where hypomethylation of subtelomeric regions was associated in the ICF1 cells with advanced telomere replication timing and elevated levels of transcripts emanating from telomeric regions. These findings may explain the abnormal telomeric phenotype observed in ICF syndrome [19].

We further evaluated the association of the RNA transcripts with significant DMPs, finding that 39.8% and 2.2% were associated to coding and non-coding genes, respectively. Interestingly, there were 4 DMPs associated to a long non-coding RNA (Fig 4B). These results indicate that specific non coding RNAs are target of DNMT3B and possibly regulated by DNA methylation. From the CpG content and neighborhood context standpoint (Fig 4C), the CpG island, which are regions with high dense number of CpGs, are the most extensively screened regions (48.6%) and are over-represented according to the design of the 450K Infinium array where CGs in islands represent only a 31% [10]. It is shown the classification according to functional genome distribution, indicating that the majority of the DMPs are located in intergenic regions (58%) leading to an over-representation of these regions regarding the design of the 450K infinium array where the percentage of probes in intergenic region is 24.6% (Fig 4D). Meanwhile, 24.3% of the DMPs correspond to promoter regions, defined as CpGs located at TSS1500, TSS200 and UTR regions. Interestingly this promoter group is under-represented based on the expected 38.9% from the 450K array [20]. Moreover, we took advantage of results published in [8] and compared the functional genomic distribution between our 181 DMPs and the 296,964 DMRs previously reported. We observed overall a discrepancy in promoter region (24.3% vs 4.5% in our study and Heyn et al, respectively) (S2B Fig, left panel). However, this striking result may be explained by the distribution of the CpGs in the array and in the whole genome that mimics our obtained pattern (S2B Fig, right panel). Finally, analyzing a subset of DMPs located in promoter regions only, we observed that 13 (87%) are associated with CpG islands or shores. Interestingly, these regions have been reported for being important regulatory regions for disease [21],[22]. As a global conclusion, the majority of the hypomethylated DMPs in ICF patients are located at intergenic and CpG islands regions.

### DNA hypomethylated candidate validation

To confirm the methylation results obtained by genome-scale techniques, we used a small scale, site-specific technique as targeted bisulfite sequencing. This technique takes advantage of the activity of sodium bisulfite that converts non-methylated cytosines into uraciles by deamination, while methyl-cytosines remain unaltered. We performed a technical validation using the same samples that were hybridized; the ICF fibroblasts and LCL (GM08747 and PT5) and 3 unrelated controls (XX, MS and 3674). Moreover, to gain robustness we also performed a biological validation using non-hybridized LCLs from the same ICF patient (GM08714) and ICF fibroblast compared with related controls (GM08728, GM08729 respectively mother and father of GM08714/GM08747) and the unrelated control (LDA). GM8714 was the ICF patient sample used in our previously paper [8].

Based on previous knowledge that epigenetic disruption of germ line function in somatic tissues has been associated with ICF [23][24], we selected four representative genes related to germ line specific pathways and/or are expressed exclusively in germ cells [25–27]. These four genes (BOLL, SYCP2, LDHAL6A and NCRNA00221) presented DNA methylation differences at crucial regulatory elements such as promoter with CpG islands. It is worth to mention that more than one DMCpGs in those important regulatory regions were identified in our analysis for BOLL and NCRNA00221, 6 and 4 respectively. Thus, coding genes such as, BOLL, SYCP2 and LDHAL6A, might be additional examples of DNMT3b target genes functionally regulated by DNA methylation in somatic tissues. Moreover, NCRNA00221 (Linc00221) is a long intergenic non-coding RNA (lincRNA). These non-coding genes are important regulators of gene expression that have been described in several diseases [28].

**Fig 5:**
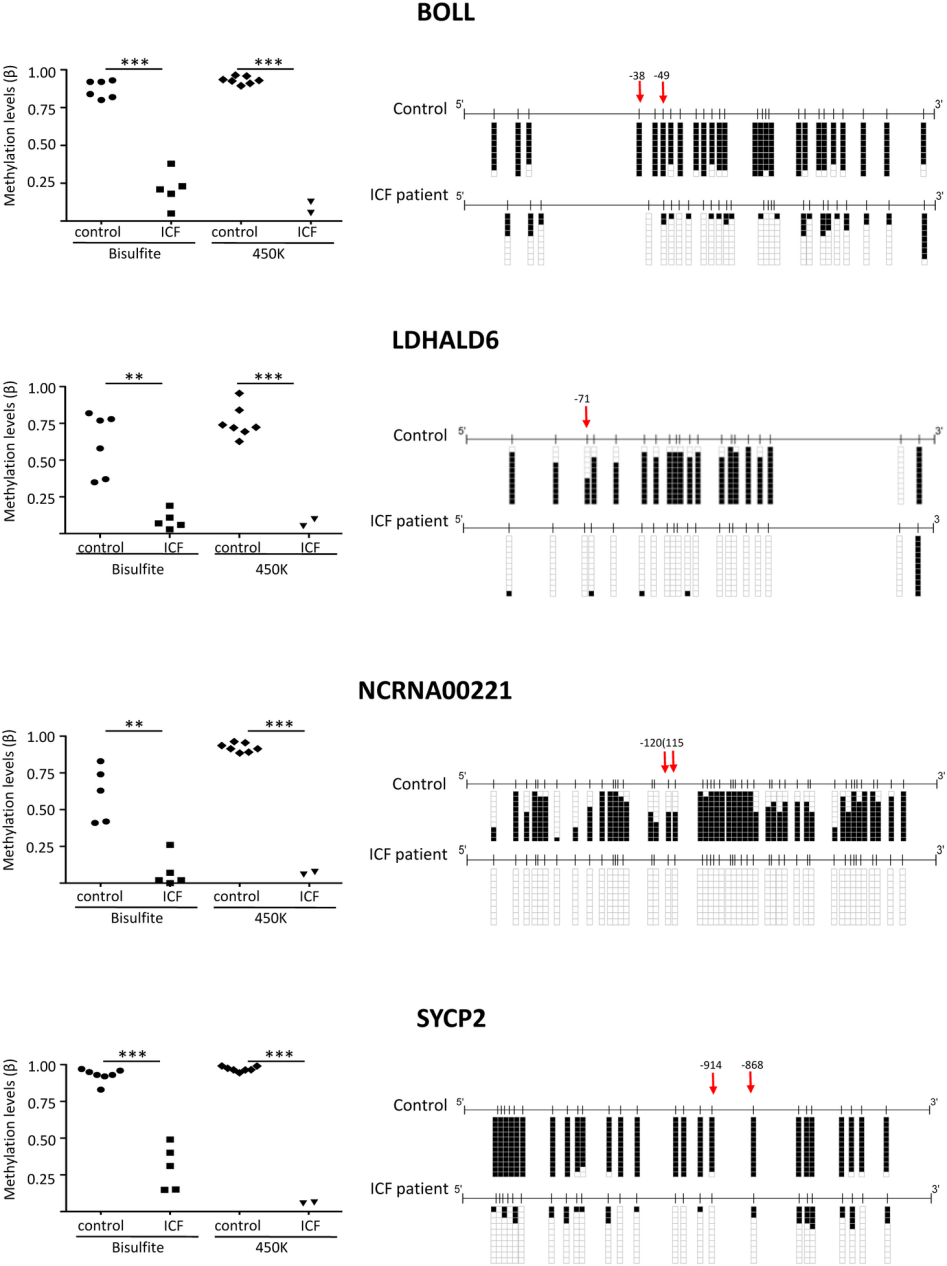
Validation of DNA methylation of four representative genes (BOLL, SYCP2, LDHALD6 and NCRNA00221) levels by bisulfite genomic sequencing analysis. Left panel depicts DNA methylation values (Beta) from ICF and healthy donors using both methodologies bisulfite genomic sequencing (BSG) and 450K array. Right panel shows bisulfite genomic sequencing analysis of genes in one representative control and ICF patient. CpG dinucleotides are shown in vertical lines. Multiple single clones are represented for each sample. Presence of unmethylated or methylated CpGs is indicated by white or black squares, respectively. Red arrows mark the localization of the differentially methylated CpGs by 450K array. The distance to transcription start site (bp) is also indicated.

The pyrosequencing analysis confirmed, by comparing ICFs patients with controls, the different methylation levels in the targeted promoter CpGs of the four genes (BOLL, SYCP2, LDHAL6A and NCRNA00221) (Fig 5, left panel). Both DNA methylation assays, bisulfite genomic sequencing and 450K infinium array, showed similar levels of methylation. Right panel of Fig 5 depicts CpGs DNA methylation values (including the CpGs obtained by 450K array analysis marked with a red arrow) located in the regions analyzed for the four genes in two representative samples: an ICF patient and a healthy control. DNA methylation values for all samples (ICF patients and control donors) and a schematic representation of the four selected gene regions are shown in (S3, S4, S5 and S6 Figs). Globally, we can conclude that not only the infinium-targeted CpGs are unmethylated in ICF patients (red arrow), but the surrounding CpGs also show the same pattern favoring the idea that global DNA demethylation landscape is maintained. This effect could indicate that the entire CpG island, and not only a single base demethylation, is altered as consequence of the DNMT3B deficiency in the ICF disease and might cause gene expression deregulation.

### Association of gene expression and DNA methylation changes

The existence of aberrations in the DNA methylation patterns of ICF cells, particularly the hypermethylation of the CpG island sequences located in the promoter regions of key regulatory genes, that lead to gene silencing, have been extensively described in literature [29]. Conversely, DNA hypomethylation has been associated mainly with DNA methylation loss at genome-wide level, although it also occurs locally. In this light, the effect of DNA demethylation makes accessible the transcription machinery and hence facilitating gene activation, which have been mainly described in cancer, involving the role of oncogenes [22]. Although some hypomethylated genes have been found associated to ICF1 [23], the number of disrupted epigenetic genes identified is very limited and little is known about their association with the etiology of this disease. In line with this finding, we aimed to evaluate the impact of DNA methylation changes on the transcriptional activity and detect gene promoters that are highly methylated in healthy controls a suffer de novo established loss-of-methylation in ICF patients concordant with gene up-regulation.

**Fig 6:**
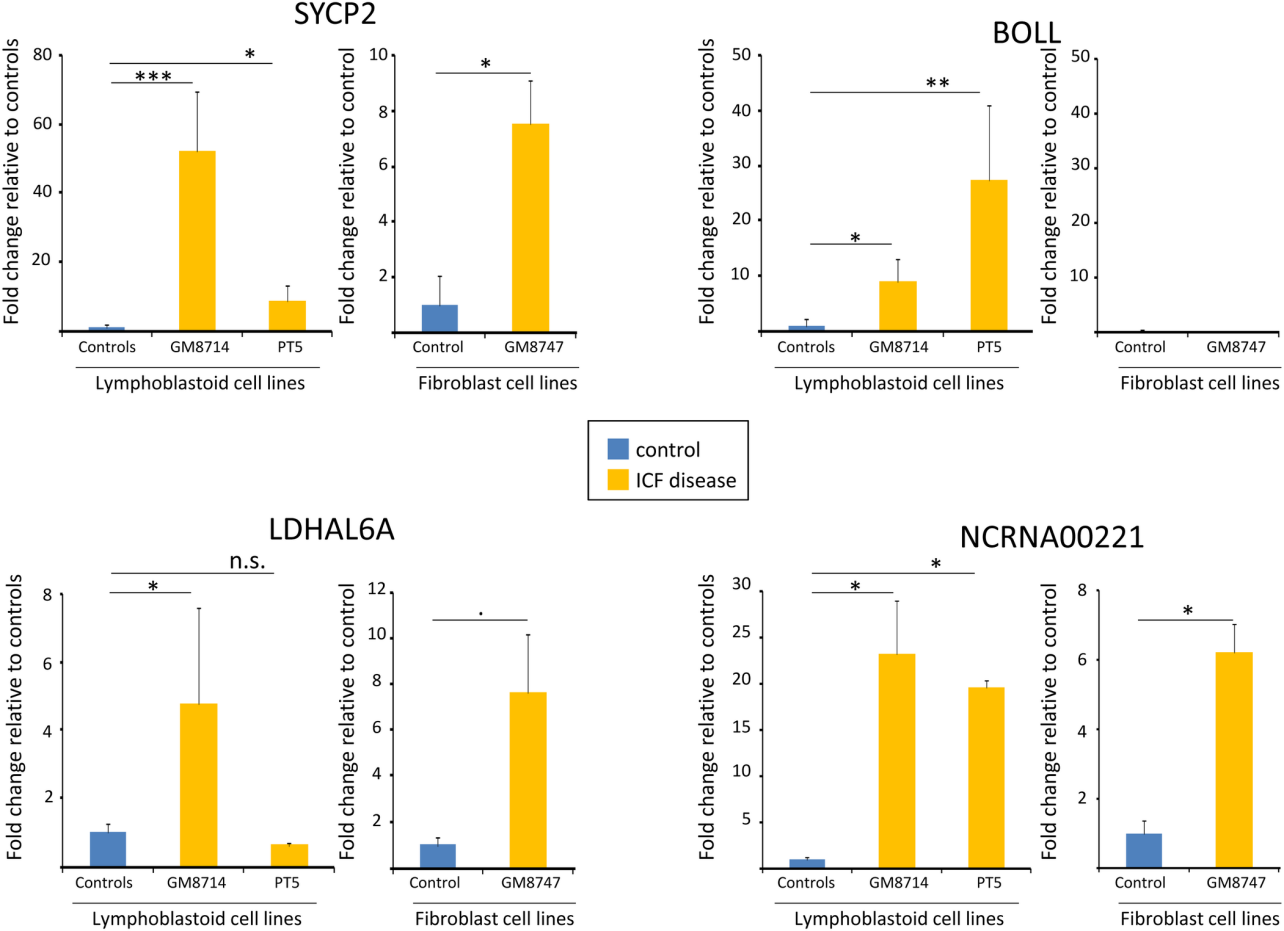
Gene expression analysis for the selected 4 CpG island-promoter associated genes BOLL, SYCP2, LDHALD6 and NCRNA00221. Fold change values of the differentially DNA methylated genes in lymphoblastoid ICF patients and healthy donors were evaluated by qRT-PCR. In parallel, Fold change values were also tested in untransformed fibroblast form an ICF patient and a healthy donor. Values were determined at least in triplicate. Statistic analysis was evaluated using student t test and significance symbols correspond to (* p<0.05; ** p<0.01 and *** p<0.001).

Therefore, based on the established idea that promoter CpG islands (CGI) are the prominent and crucial regulatory regions for gene expression and with high variable methylation level between normal and diseased tissue [30],[31], we sought to evaluate gene expression of those previously validated genes. To elucidate the impact of DNA methylation on the transcriptional activity of SYCP2, BOLL, LDHAL6A and NCRNA00221, we analyzed by qPCR their gene expression in ICF patient cells (GM08714 and PT5) and control samples including related and unrelated healthy donors (GM08728, GM08729, MS and LDA). We also analyzed in parallel the untransformed fibroblasts form ICF patient (GM08747) and the healthy donor (GM03674). The results clearly showed a significant up-regulation, using the unpaired Student t test, for three of four genes in ICF cells (except LDHAL6A that only show significance in GM08714, but not in PT5) compared to controls, indicating that the impaired DNA methylation at the identified DMPs is critical for controlling their gene activity (Fig 6). Differences in expression levels between both ICF samples in SYCP2 and BOLL could be due to DNMT3 type of mutation and the idea that, the other factors together with DNA methylation changes may be involved in the complex regulation of gene expression. In line, consistent results were obtained in fibroblasts expression analysis, except for BOLL that we were not able to detect expression in any fibroblast. This could be explained due to a tissue-specific regulation beyond DNA methylation. Interestingly, even if at different extents overall comparing levels in LCLs with untransformed fibroblasts, three genes were epigenetically regulated in ICF patients with four different DNMT3B mutations (GM8714, PT5). This suggests an important role in the DNMT3B-mediated regulatory pathway that could contribute to explain some aspects of the characteristic phenotype of these ICF patients. These results suggest a potential interaction between the DNMT3B type of mutation and the epigenetic regulation and intensity of gene activation of the studied genes.

The role of DNMT3b in protecting somatic cells against the aberrant expression of the germ line program has recently been suggested [23]. Moreover, the DNMT3b-mediated silencing of a subset of germ line genes in somatic cells occurs through the recruitment of the E2F6 transcriptional repressor at their promoter region [32]. In this light, by identifying novel germ line genes, which are hypomethylated and inappropriately expressed, our results suggest that this phenomenon in the context of DNMT3B deficiency might be rather widespread. How this specific deregulation may contribute to ICF molecular pathogenesis remains to be established. Notably, the ectopic expression of meiotic genes in cancer cells has been functionally related to abnormal chromosome segregation and aneuploidy [33]. Because chromosomal instability is a hallmark of ICF syndrome, this raises the possibility that loss of silencing at particular germ line genes drives the typical cytological abnormalities seen in ICF patient lymphocytes.

It is known that early diagnosis of ICF syndrome is crucial since early treatment can improve the course of disease. However, ICF is probably underdiagnosed, especially in patients that present incomplete phenotype or born to families with no affected relatives [23]. Therefore, the DNA hypomethylation profile of NCRNA00221 especially, and in a minor extent SYCP2 and BOLL, could be further investigated and validated in peripheral blood in order to develop specific clinical biomarkers to facilitate the identification of ICF patients.

## Conclusion

Our results contribute to elucidate how different mutations in DNMT3b result in deficiency of DNA methyltransferase activity, eventually causing ICF1 syndrome. It seems accepted that is the DNA methylation deficiency, and not other aspects of impaired DNMT3b activity, responsible for the ICF syndrome.

The regions with aberrant methylation in ICF patients were almost exclusive of pericentromeric regions of chromosome 1, 16, sometimes 9 and associated to repeated DNA sequences or heterochromatic genes. Although in vitro studies identified a spectrum of biochemical defects in the catalytic function associated to ICF-specific DNMT3B mutations, their impact on the genome-wide DNA methylation level in patient-derived cells is unsolved. Our results contributed to characterize the global defects of DNA methylation pattern in two heterozygous DNMT3B-mutant backgrounds, uncovering novel ICF-specific hypomethylated sites, outside the pericentromeric regions and in other chromosomes compared to those previously mentioned. Interestingly, we identified additional DNMT3B target loci whose expression must be restricted to germ cells. Establishment and maintenance of promoter DNA methylation in somatic tissues by DNMT3B is critical for their transcriptional repression. In addition, to provide further evidence on DNMT3B role in silencing germ line genes, these findings are of particular interest in the context of other human disease, like cancer. It is remarkable that the expression of catalytically inactive DNMT3b splice variants, the aberrant transcription of germ line genes and chromosomal instability are shared features. We thus believe that these genome-wide studies will help to elucidate the relationship between DNA hypomethylation and pathological phenotypes.

Finally, from the ICF syndrome point of view, our results contribute to further evaluate the utility of these potential biomarkers as diagnostic markers.

## Supporting Information

S1 Fig Individual genome-wide DNA methylation profiles in ICF patients and control samples. (A) Histograms showing bimodal distribution pattern of DNA methylation profiles in the two ICF patients (PT5 and GM8748) and normal donors (XX, MS and GM3674). (B) Table showing number of poorly methylated and highly methylated for each sample hybridized (C) Individual scatter plots combining ICF patients and normal donors. (D) Individual box plots for ICF patients compared to normal donors. Normality was tested using the Shapiro-Wilk test and significance was evaluated with the Mann-Whitney U test and is indicated by three asterisks *** (p<0.001).
(TIF)

S2 Fig Comparison of Hypomethylated regions reported by Heyn et al. and current differentially methylated positions. (A) Venn diagram illustrating the number of hypomethylated features. The intersecting region represents those CpGs that are common to both analyses. (B) Left panel: graph depicting percentages of differentially methylated features based on functional genomic distribution comparing both analyses (dark blue for our analysis and dark red for Heyn et al.). Right panel: graph showing percentages based on the 450K array design and the whole genome (light blue for 450K array and red for whole genome). Data obtained from [20].
(TIF)

S3 Fig Bisulfite genomic sequencing for technical and biological validation of differentially DNA methylated gene BOLL. For technical validation two ICF (PT5 and GM08747) and two unrelated controls (XX, MS and GM03674) were used. For biological validation two ICF patient samples (GM8714 and PT3) and three controls (two related controls GM8728, GM8729 and one unrelated control LDA) were analyzed. CpG dinucleotides is shown in vertical lines. Multiple single clones are represented for each sample. Presence of unmethylated or methylated CpGs is indicated by white or black squares, respectively. Red arrows mark the localization of the differentially methylated CpGs by 450K array. The distance to Trasncription Start Site (bp) is also indicated.
(TIF)

S4 Fig Bisulfite genomic sequencing for technical and biological validation of differentially DNA methylated gene SYCP2. The design is similar to S3 Fig.
(TIF)

S5 Fig Bisulfite genomic sequencing for technical and biological validation of differentially DNA methylated gene LDHAL6A. The design is similar to S3 Fig.
(TIF)

S6 Fig Bisulfite genomic sequencing for technical and biological validation of differentially DNA methylated gene NCRNA00221. The design is similar to S3 Fig.
(TIF)

S1 Table Primers for bisulfite genomic sequencing. (DOCX)

S2 Table Primers for quantitative RT-PCR. (DOCX)
