# Supplementary figures and images for: Genome-Wide DNA Methylation Analysis Identifies Novel Hypomethylated Non-Pericentromeric Genes with Potential Clinical Implications in ICF Syndrome
Source: PLoS One. 2015 Jul 10;10(7):e0132517. doi: 10.1371/journal.pone.0132517 (PMC4498748; doi:10.1371/journal.pone.0132517)

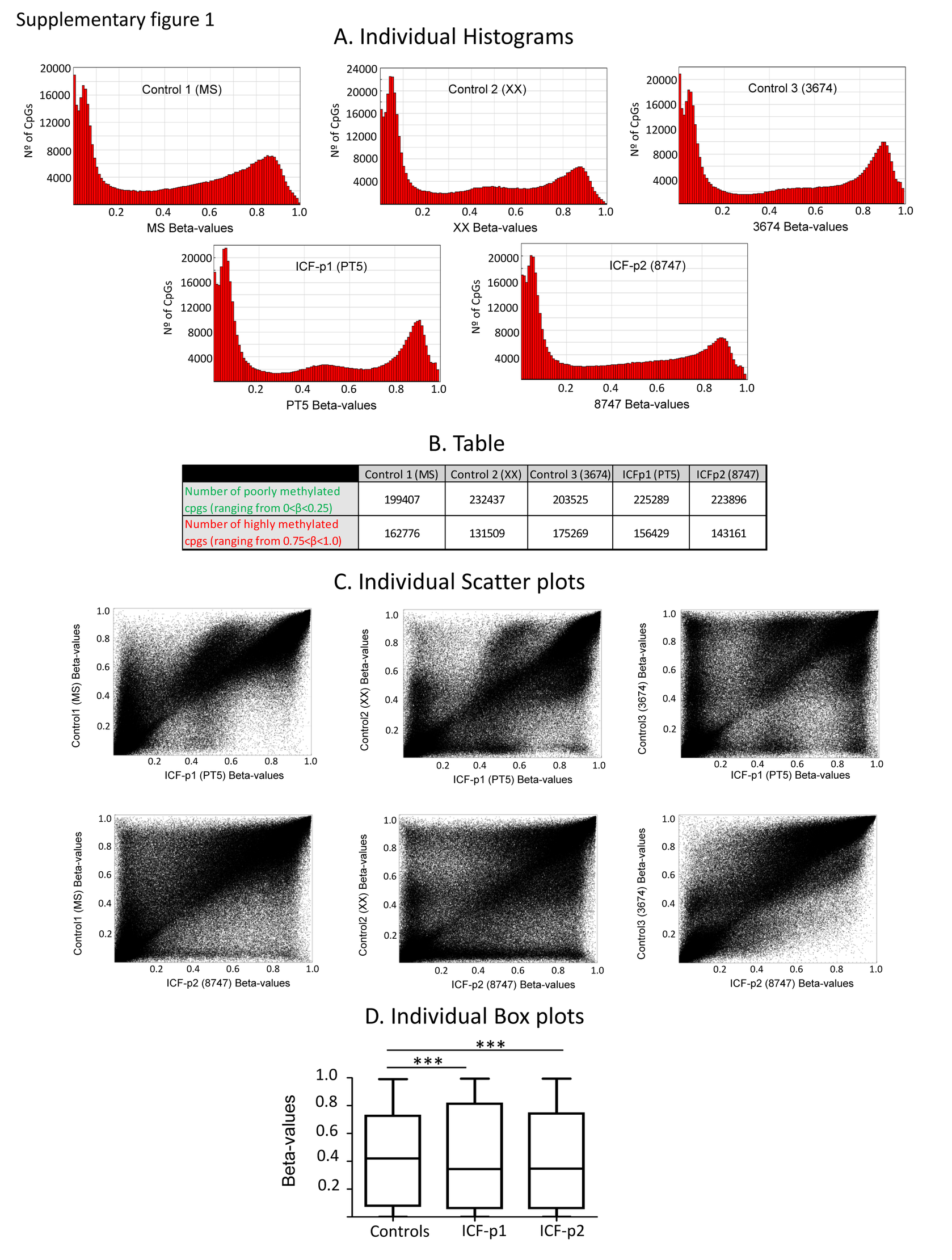

Supplement: S1 Fig — (A) Histograms showing bimodal distribution pattern of DNA methylation profiles in the two ICF patients (PT5 and GM8748) and normal donors (XX, MS and GM3674). (B) Table showing number of poorly methylated and highly methylated for each sample hybridized (C) Individual scatter plots combining ICF patients and normal donors. (D) Individual box plots for ICF patients compared to normal donors. Normality was tested using the Shapiro-Wilk test and significance was evaluated with the Mann-Whitney U test and is indicated by three asterisks *** (p<0.001). (TIF) [file pone.0132517.s001.tif]

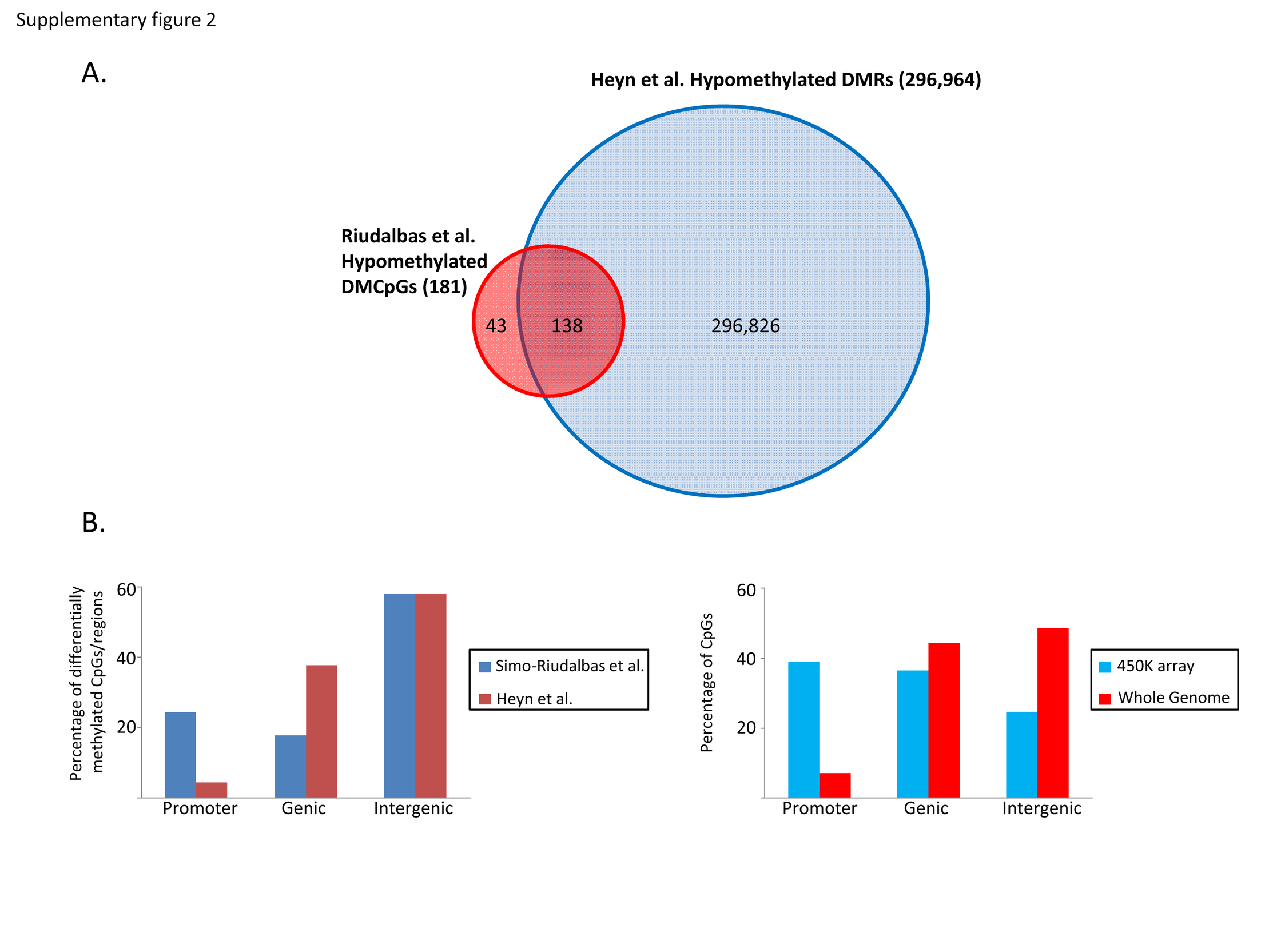

Supplement: S2 Fig — (A) Venn diagram illustrating the number of hypomethylated features. The intersecting region represents those CpGs that are common to both analyses. (B) Left panel: graph depicting percentages of differentially methylated features based on functional genomic distribution comparing both analyses (dark blue for our analysis and dark red for Heyn et al.). Right panel: graph showing percentages based on the 450K array design and the whole genome (light blue for 450K array and red for whole genome). Data obtained from [20]. (TIF) [file pone.0132517.s002.tif]

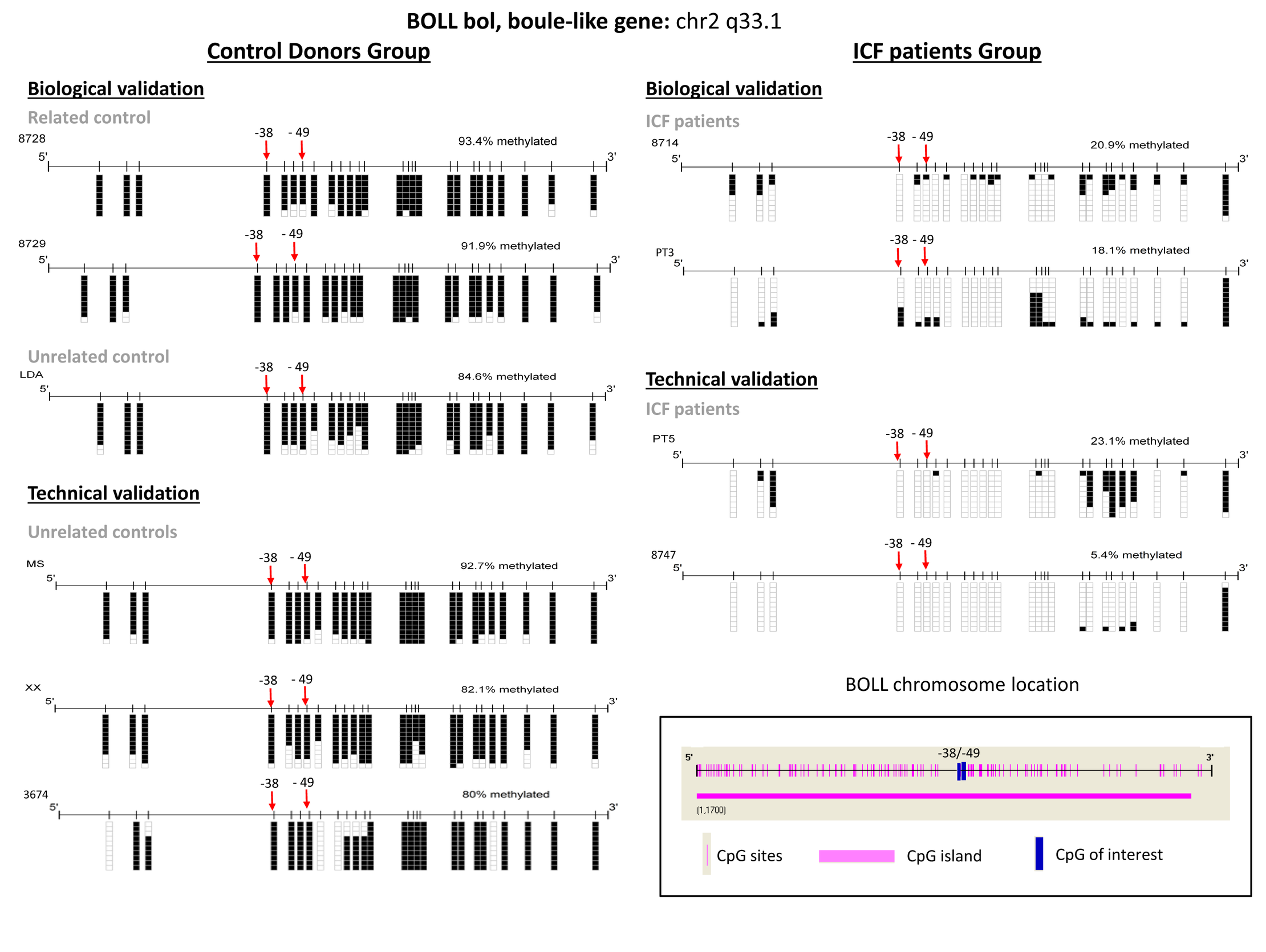

Supplement: S3 Fig — For technical validation two ICF (PT5 and GM08747) and two unrelated controls (XX, MS and GM03674) were used. For biological validation two ICF patient samples (GM8714 and PT3) and three controls (two related controls GM8728, GM8729 and one unrelated control LDA) were analyzed. CpG dinucleotides is shown in vertical lines. Multiple single clones are represented for each sample. Presence of unmethylated or methylated CpGs is indicated by white or black squares, respectively. Red arrows mark the localization of the differentially methylated CpGs by 450K array. The distance to Trasncription Start Site (bp) is also indicated. (TIF) [file pone.0132517.s003.tif]

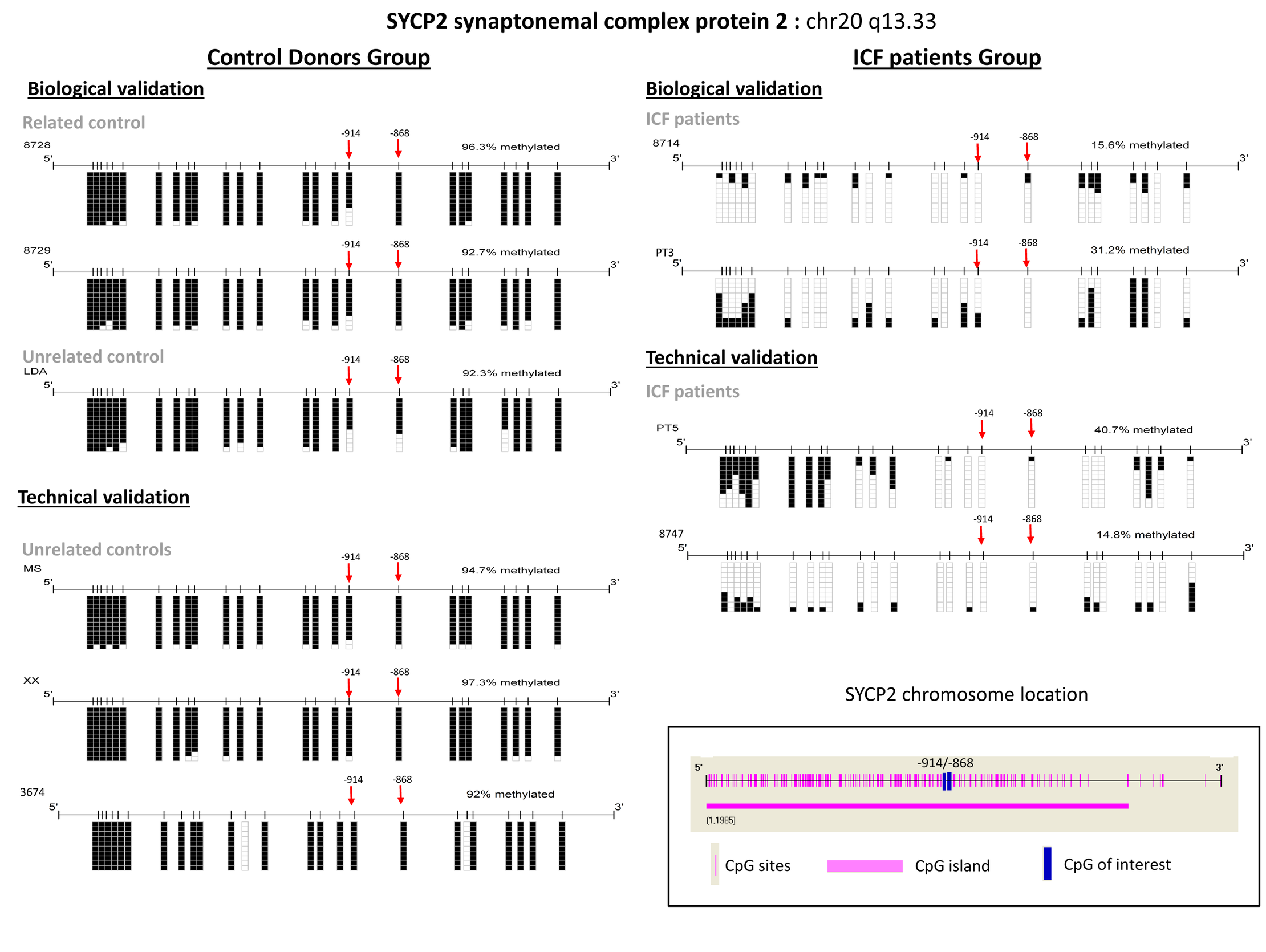

Supplement: S4 Fig — The design is similar to S3 Fig. (TIF) [file pone.0132517.s004.tif]

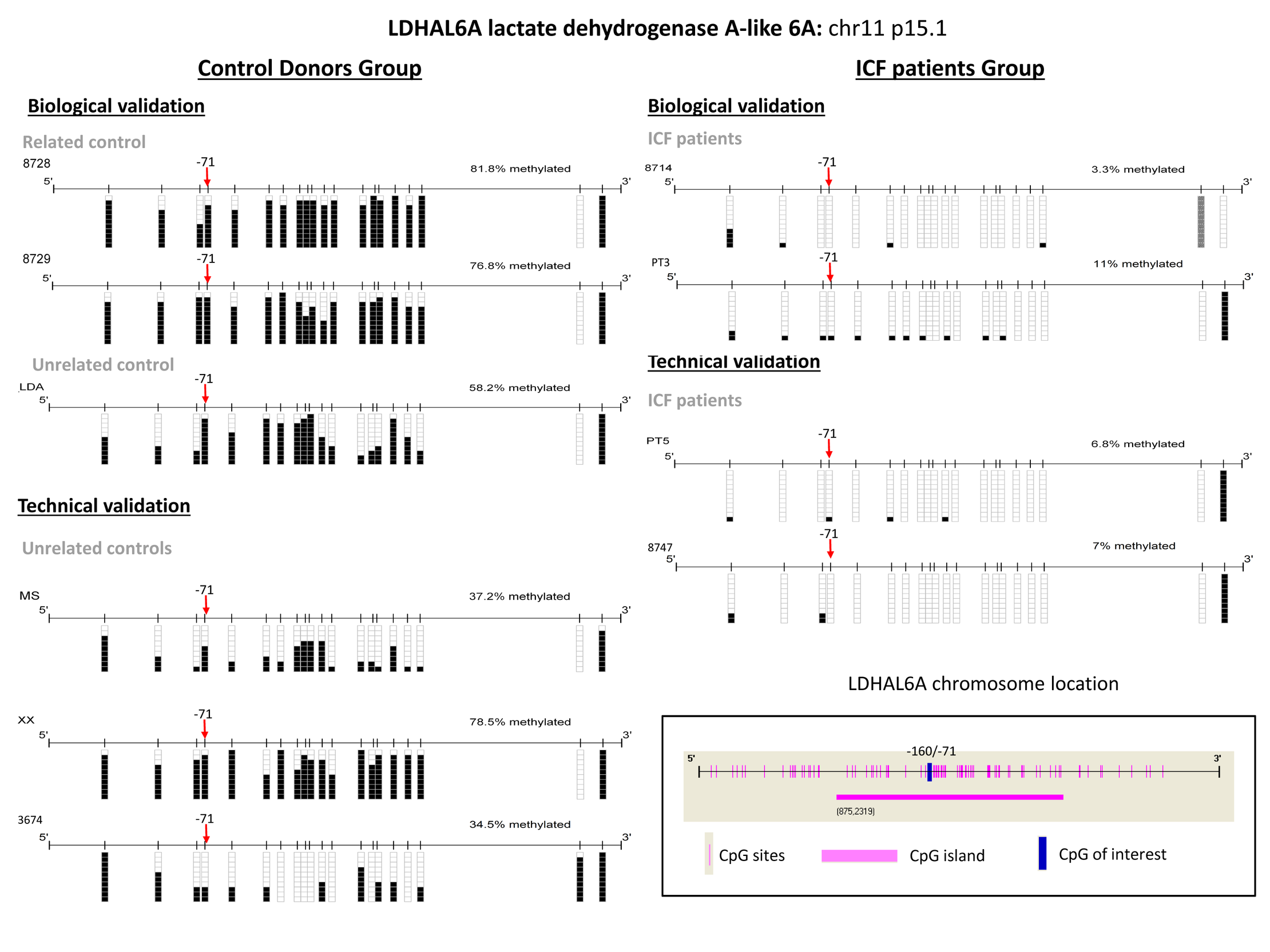

Supplement: S5 Fig — The design is similar to S3 Fig. (TIF) [file pone.0132517.s005.tif]

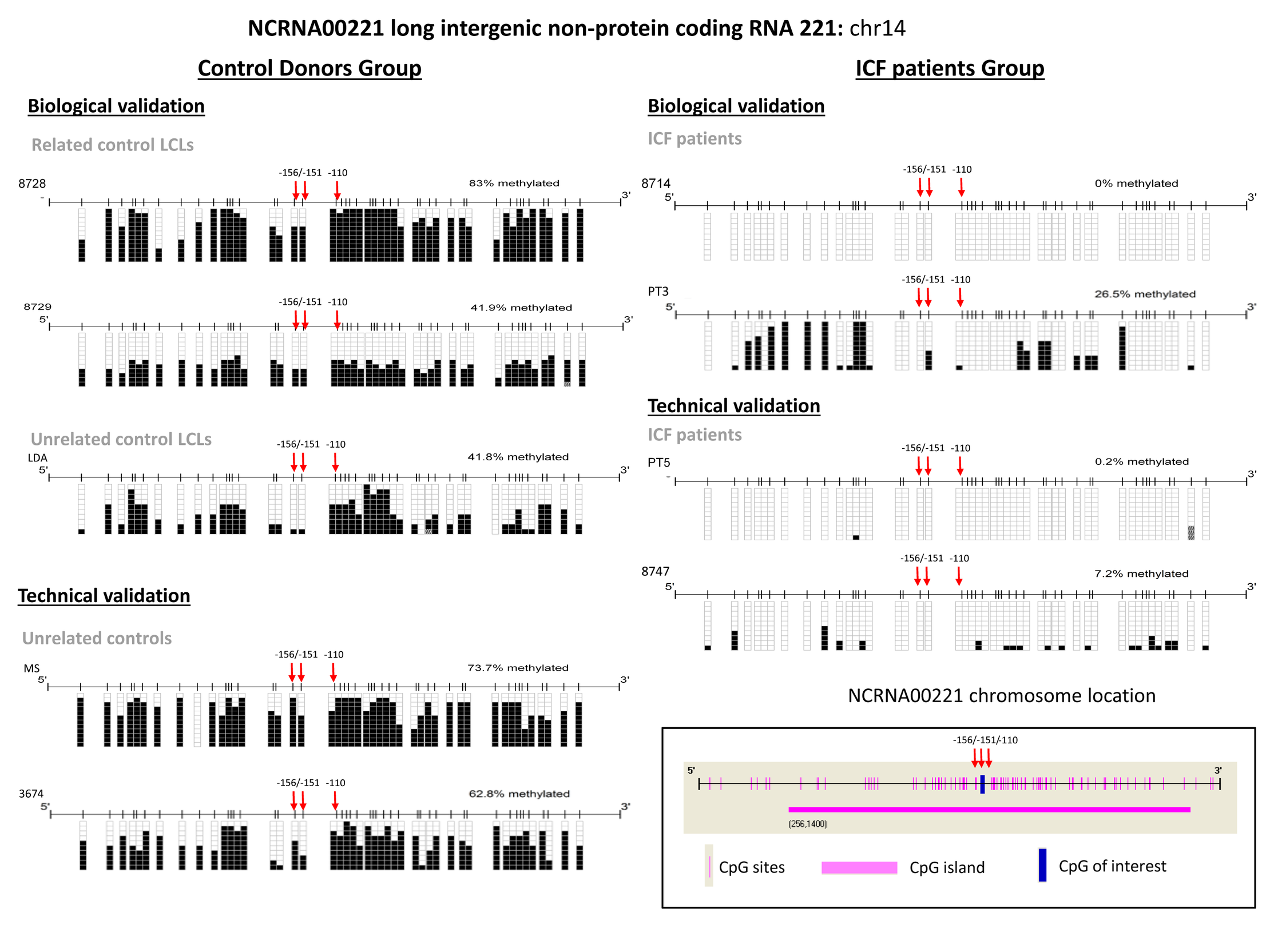

Supplement: S6 Fig — The design is similar to S3 Fig. (TIF) [file pone.0132517.s006.tif]
