# Supplementary material for: Genome-Wide DNA Methylation Analysis Identifies Novel Hypomethylated Non-Pericentromeric Genes with Potential Clinical Implications in ICF Syndrome
Source: PLoS One. 2015 Jul 10;10(7):e0132517. doi: 10.1371/journal.pone.0132517 (PMC4498748; doi:10.1371/journal.pone.0132517)
Supplement: S1 Table — (DOCX) [file pone.0132517.s007.docx]

Supplementary table 1. Primers for bisulfite genomic sequencing

| **Name** | **Sequence (5´-3´)** | **Length** |
| --- | --- | --- |
| BOLL-BSP-F | GGAAGGTTTGAGAGATTTGGTA | 22 |
| BOLL-BSP-R | AACCAACRAAAAATTTTACCC | 21 |
| LDHAL6A-BSP-F | GTGGATGGGTTTTAGGTAAATAA | 23 |
| LDHAL6A-BSP-R | ACCCAACTCRTACAAACATC | 20 |
| NCRNA00221_BSP_F | GGTTGTATGGTTTGAAGAGAAG | 22 |
| NCRNA00221_BSP_R | CCAACCCTATACCACATAAAAA | 22 |
| SYCP2-BSP-F | GGGAGTAGGTTTGGGGTT | 18 |
| SYCP2-BSP-R | CACTCCCTTCTTCCCTAAATAA | 22 |
