# Supplementary material for: Genome-Wide DNA Methylation Analysis Identifies Novel Hypomethylated Non-Pericentromeric Genes with Potential Clinical Implications in ICF Syndrome
Source: PLoS One. 2015 Jul 10;10(7):e0132517. doi: 10.1371/journal.pone.0132517 (PMC4498748; doi:10.1371/journal.pone.0132517)
Supplement: S2 Table — (DOCX) [file pone.0132517.s008.docx]

Supplementary table 2. Primers for quantitative RT-PCR

| **Name** | **Sequence (5´-3´)** | **Length** |
| --- | --- | --- |
| BOLL F | TGATGGCACTTGGAGCATAA | 20 |
| BOLL R | AATTGCACAGGATGGTGGAT | 20 |
| LDHAL6A F | CACGCCTTGATTTAGTCCAGC | 21 |
| LDHAL6A R | CCACTCAACTTCCAGGCTACA | 21 |
| LINC00221 F | GGTAGCTCAGCGGGGACTT | 19 |
| LINC00221 R | CTCTCCCAGCCAGGACCTC | 19 |
| SYCP2 F | ACATGCCCATTTGCTGTGTA | 20 |
| SYCP2 R | TGTTGAATTCCCAAACCAAAA | 21 |
